# Recreational Fish-Finders—An Inexpensive Alternative to Scientific Echo-Sounders for Unravelling the Links between Marine Top Predators and Their Prey

**DOI:** 10.1371/journal.pone.0140936

**Published:** 2015-11-23

**Authors:** Alistair M. McInnes, Arjun Khoosal, Ben Murrell, Dagmar Merkle, Miguel Lacerda, Reason Nyengera, Janet C. Coetzee, Loyd C. Edwards, Peter G. Ryan, Johan Rademan, Jan J van der Westhuizen, Lorien Pichegru

**Affiliations:** 1 Percy FitzPatrick Institute, DST/NRF Centre of Excellence, University of Cape Town, Cape Town, South Africa; 2 Computational Biology Group, Institute of Infectious Diseases and Molecular Medicine, University of Cape Town, Cape Town, South Africa; 3 Department of Medicine, University of California San Diego, La Jolla, California, United States of America; 4 Branch: Fisheries Management, Department of Agriculture, Forestry and Fisheries, Rogge Bay, South Africa; 5 Department of Statistical Sciences, University of Cape Town, Cape Town, South Africa; 6 DST/NRF Centre of Excellence at the Percy FitzPatrick Institute of African Ornithology, Department of Zoology, Nelson Mandela Metropolitan University, Port Elizabeth, South Africa; 7 Raggy Charters, Port Elizabeth, South Africa; Centre National de la Recherche Scientifique, Centre d'Etudes Biologiques de Chize, FRANCE

## Abstract

Studies investigating how mobile marine predators respond to their prey are limited due to the challenging nature of the environment. While marine top predators are increasingly easy to study thanks to developments in bio-logging technology, typically there is scant information on the distribution and abundance of their prey, largely due to the specialised nature of acquiring this information. We explore the potential of using single-beam recreational fish-finders (RFF) to quantify relative forage fish abundance and draw inferences of the prey distribution at a fine spatial scale. We compared fish school characteristics as inferred from the RFF with that of a calibrated scientific split-beam echo-sounder (SES) by simultaneously operating both systems from the same vessel in Algoa Bay, South Africa. Customized open-source software was developed to extract fish school information from the echo returns of the RFF. For schools insonified by both systems, there was close correspondence between estimates of mean school depth (R^2^ = 0.98) and school area (R^2^ = 0.70). Estimates of relative school density (mean volume backscattering strength; S_v_) measured by the RFF were negatively biased through saturation of this system given its smaller dynamic range. A correction factor applied to the RFF-derived density estimates improved the comparability between the two systems. Relative abundance estimates using all schools from both systems were congruent at scales from 0.5 km to 18 km with a strong positive linear trend in model fit estimates with increasing scale. Although absolute estimates of fish abundance cannot be derived from these systems, they are effective at describing prey school characteristics and have good potential for mapping forage fish distribution and relative abundance. Using such relatively inexpensive systems could greatly enhance our understanding of predator-prey interactions.

## Introduction

### Predator-prey interactions in the marine environment

Predator-prey interactions are central to ecosystem functioning and shape species evolution [1,2]. Recent technological developments have greatly improved our understanding of ecosystem functioning and animal behaviour, especially in the marine environment, where remote sensing and data logging technologies have revolutionized the collection of ecological data [3–5]. Numerous studies have used biotelemetry, e.g. data-loggers, attached to marine top predators to gather information on their habitat use and response to ocean physical processes [3,6]. Combining these data with diet studies and/or remote sensing of oceanographic covariates provides insights into prey availability and ecosystem functioning [7–11]. However, relatively few studies have been able to assess predator responses in terms of fish prey distribution and abundance (see [12] for a review on seabirds). Such studies generally are over large spatial scales, which often results in a mismatch between prey and predator distributions, e.g. [13]. Far-ranging species occupy a relatively predictable environment with clear associations between prey and oceanic features [14]. However, many marine top predators (especially central place foragers such as breeding seabirds and seals) occupy a relatively small home-range (at least seasonally) within systems that exhibit great variability in prey abundance. Unlike physical processes that, thanks to advances in satellite, mooring and biotelemetry technology, have become increasingly easy to obtain at fine spatio-temporal scales, data on the distribution and abundance of prey remains costly to gather. This is largely due to the specialised nature and application of the surveys, i.e. typically to quantify fish stocks for the setting of quotas, the associated large spatio-temporal scales of study and the costly nature of these operations. Consequently, this lack of prey distribution data beyond the scales of conventional applications remains a serious impediment to marine ecology studies.

### Monitoring prey availability for African Penguins–a case study

The African Penguin (*Spheniscus demersus)* feeds almost exclusively on pelagic fish species, predominantly sardine (*Sardinops sagax*) and anchovy (*Engraulis capensis*), that are also targeted by industrial fisheries [15]. The population of African Penguins has decreased dramatically over the last 12 years, resulting in its conservation status being raised to ‘Endangered’ [16]. Several studies have suggested that decreased localised prey abundance is driving this trend [17–19], prompting an assessment of the impacts of purse-seine fishing on foraging and breeding parameters of these birds by temporarily excluding fishing around selected breeding colonies. Results to date include a significant positive relationship between penguin foraging effort and purse-seine catches in Algoa Bay [20,21] although the earlier results were disputed by [22] who claimed that the study did not account for natural fluctuations in prey abundance. To address this shortcoming, fine-scale (temporal and spatial) pelagic fish surveys were initiated in Algoa Bay around two of the largest African Penguin breeding colonies in 2011.

### Recreational fish-finders: an inexpensive alternative

Due to the prohibitive cost of scientific echo-sounders, we used a recreational fish-finder (RFF) designed to monitor fish in real time mostly to locate favourable fishing grounds. They are not calibrated and hence do not allow for accurate measurements of fish density and biomass as the standard performance characteristics of the system cannot be checked or monitored over time and the reference system sensitivity cannot be established. In contrast to RFFs, scientific echo-sounders (SES) are calibrated frequently/regularly with a standard target sphere with known acoustic scattering properties to determine the transducer directional and response output and receiver sensitivity [23]. This allows for the determination of fish density if the target strength (TS) of the fish species insonified is known. Other advantages of SES systems include a larger dynamic range and a higher signal to noise ratio.

The use of SESs is invariably associated with hydro-acoustic data-processing software that utilises echo-integration algorithms to compute the mean density of fish and extract quantifiable school descriptors [24]. A lack of similar software for RFFs is a serious drawback to using these systems for scientific purposes. We developed an open source hydroacoustic data-processing software for use with a Furuno DFF3 RFF, then conducted a pelagic fish survey in Algoa Bay using both RFF and SES systems on the same vessel to compare school descriptors and density estimates. We validated our approach and demonstrated the suitability of processed RFF data to marine top predator and prey interactions and fishery-related research, using the African Penguin as a case study.

## Methods

### Fish-finder software (FISH)

**Fig 1:**
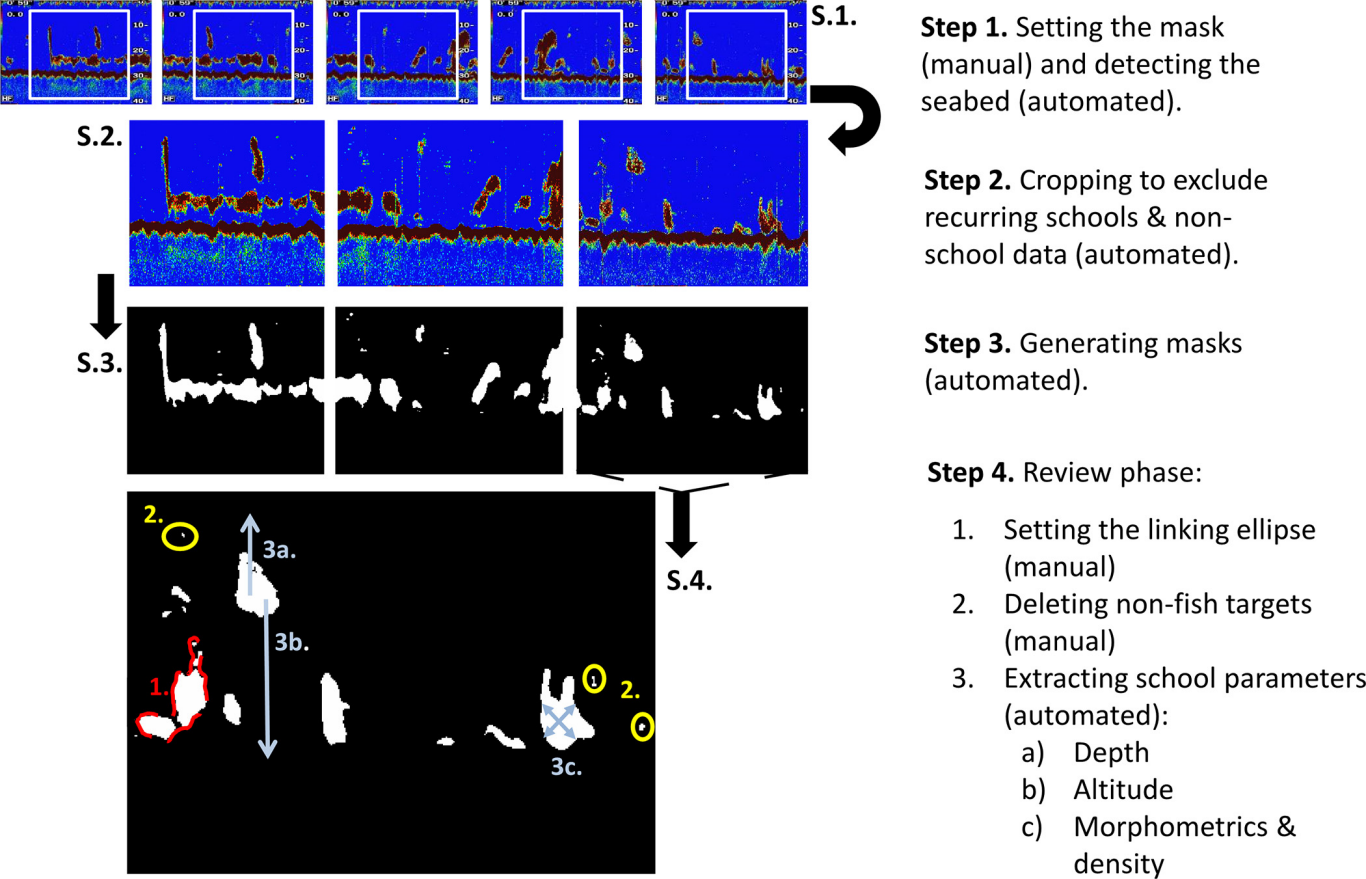
Flow diagram illustrating the workflow of FISH software showing the different steps in the processing phase (steps 1–3) and the review phase (step 4).

To analyse fish data, represented as pixels in a.png format, from our Furuno DFF3 Fish-finder, we developed the Fish-finder Image Segmentation Helper (FISH) programme, written in Java as a plugin to Fiji [25], an open source image processing platform. Two plugins are used: a processor (FISHproc) and a reviewer (FISHrev). FISHproc requires manual designation of the analysis window (Fig 1, step 1–3). Once the window is set, duplicated regions are removed from overlapping contiguous frames. Thereafter, each frame is resized, reconciling the horizontal and vertical scales. In order to extract meaningful signal, several forms of interfering noise are mitigated. First, the spurious signal arising from beneath the seabed must be excluded. To identify the seabed, we use an edge-detection filter to identify the upper edges of all objects, and then find a path that horizontally spans the frame, maximizing the path’s occupancy of the detected upper edges while minimizing vertical jumps (with a tunable anti-vertical penalty parameter). This is achieved with a dynamic programming algorithm (analogous to the Viterbi algorithm): O(N*(2P+1)), where O is the asymptotic notation, N is the pixel width of the frame, and P is the maximum allowed vertical transition per horizontal change (in our implementation, P = 2). This strategy is robust to noise that introduces spurious gaps in the sea bed. Next, speckles of interference are removed by passing each frame through a median filter, and vertical noisy columns are identified as peaks in the echo returns and subsequently removed. The final step of the initial processing phase involves the generation of masks of echo-returns from these previous steps. The second plugin, FISHrev, is used in a post-processing review phase and for subsequent automated feature extraction (Step 4, see Fig 1). During the review phase, the user specifies the dimensions of the linking ellipse to define the encompassing area of an aggregated school. This stage enables the user to then scroll through each image with the ability to toggle between the mask or raw image mode to delete unwanted anomalies, e.g. noise, dispersed fish layers or school-like bathymetric features. At the end of the review phase FISHrev extracts school parameters to a.csv output file (Table 1).

**Table 1:** School parameters and their descriptions for Fish-finder Image Segmentation Helper (FISH) outputs.

Parameter	Description
File	Source file name
Time	HH:MM:SS
Date	YY/MM/DD
PicRef	File name of picture
Lat_dd	Latitude in decimal degrees
Long_dd	Longitude in decimal degrees
LeftLat	Latitude of left extent of school
LeftLon	Longitude of left extent of school
RightLat	Latitude of right extent of school
RightLon	Longitude of right extent of school
BotAltitude (m)	Altitude at bottom of school
TopDepth (m)	Depth at top of school
MeanAltitude (m)	Mean altitude of school
MeanDepth (m)	Mean depth of school
BotDepth (m)	Depth of sea floor
SchoolHeight (m)	Vertical extent of school
SchoolWidth (m)	Horizontal extent of school
Area (m^2^)	Area of school
AreaLV (m^2^)	Area of school less vacuoles
Perimeter (m)	Perimeter of school
MaxCalDiam (m)	Length of maximum diameter
MinCalDiam (m)	Length of widest point perpendicular to maximum diameter
MaxCalAngle (°)	Angle of maximum calibrated diameter
Pixel value count	Number of pixels for each pixel type*

*Recreational fish-finders (RFF) typically classify pixels on a sequential numeric scale with no reference to actual dB values. These need to be calibrated to a scientific echo-sounder (SES).

### Inter-calibration procedures

**Fig 2:**
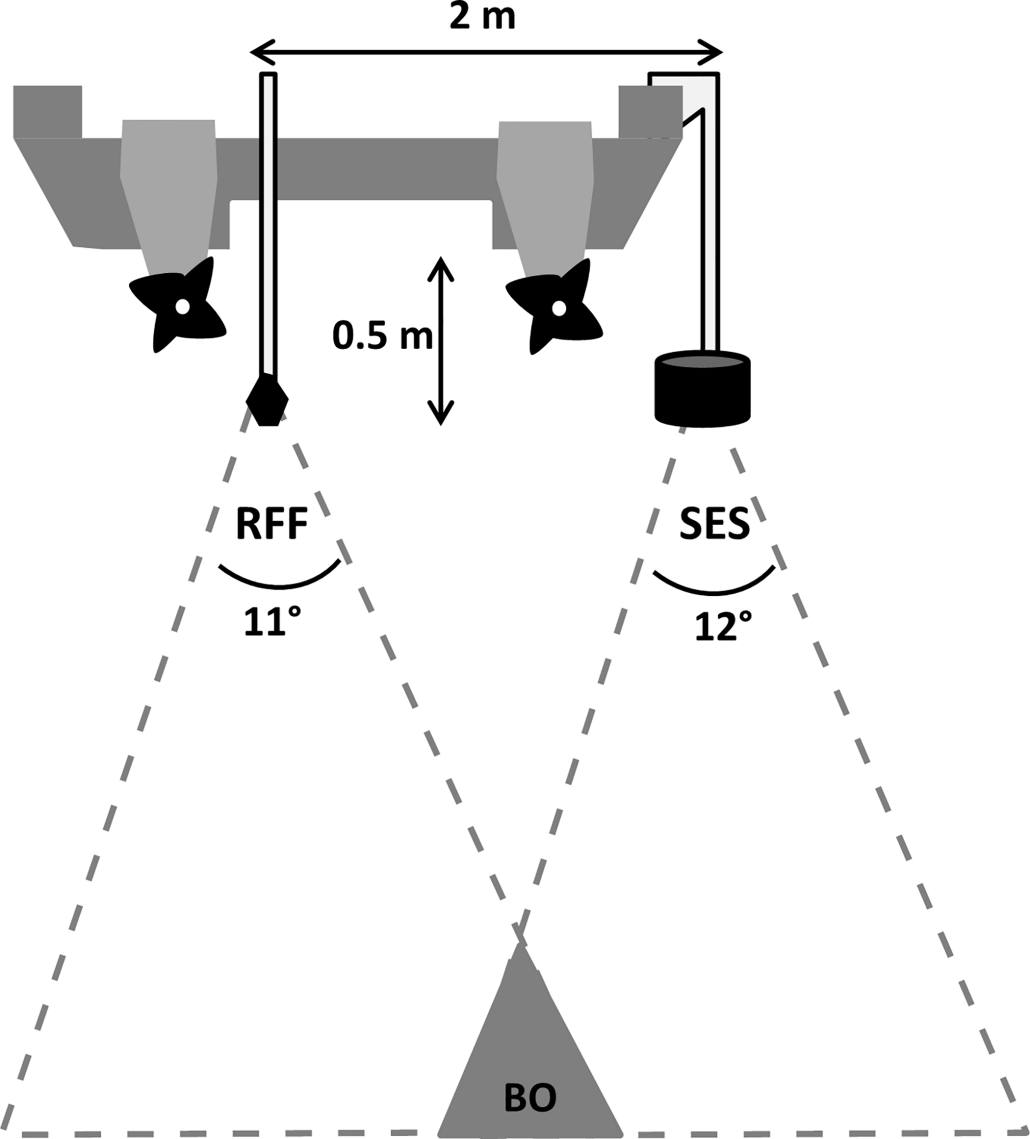
Transducer placements on catamaran ski-boat (profile view of stern) for the recreation fish-finder (RFF) and the scientific echo-sounder (SES) showing athwartship displacement (2 m), transducer depths (0.5 m), beam angles and area of beam overlap (BO), not to scale.

In May 2014, both the RFF and a Simrad EK60 38kHz SES were deployed on a 8.6 m catamaran ski-boat in Algoa Bay, on the South African south coast. The RFF transducer was attached to a 1-m stainless steel pole mounted to the stern of the boat and the SES transducer was mounted to the starboard side of the boat approximately 2 m athwartship from the RFF transducer (Fig 2). The distance between the two transducers creates the possibility that different schools could be insonified by the outer edges of the echo beams, or that a school or part thereof will not fall within the beams of both transducers, particularly at shallow depths, so perfect concordance is not expected (Fig 2). Specifications for both systems are given in Table 2. Beam angles for both transducers are similar but the RFF has a variable ping rate dependent on the depth range. There is also a notable difference in frequencies between the two systems: RFF = 200 kHz versus SES = 38 kHz.

**Table 2:** Specifications of the scientific echo-sounder and the recreational fish-finder.

Boat and sounder details	Echo-sounder	Recreational Fish-finder
Transducer mount	side	transom
Transducer depth	0.5 m	0.5 m
Transducer	Simrad ES38-12	Furuno 525TID-PWD
**Settings**		
Frequency	38 kHz	200 kHz
Gain	21.22 dB	default
Time varied gain (TVG)		2
Sa correction	-0.67 dB	none
3dB beam angle	12°	11°
Power	1000 w	600 w
Receiver band width	2.41 kHz	not specified
Max ping range	250 m	206 m
Ping rate	2–5 s^-1^	4–10 s^-1^ (100 m—30 m range)

**Fig 3:**
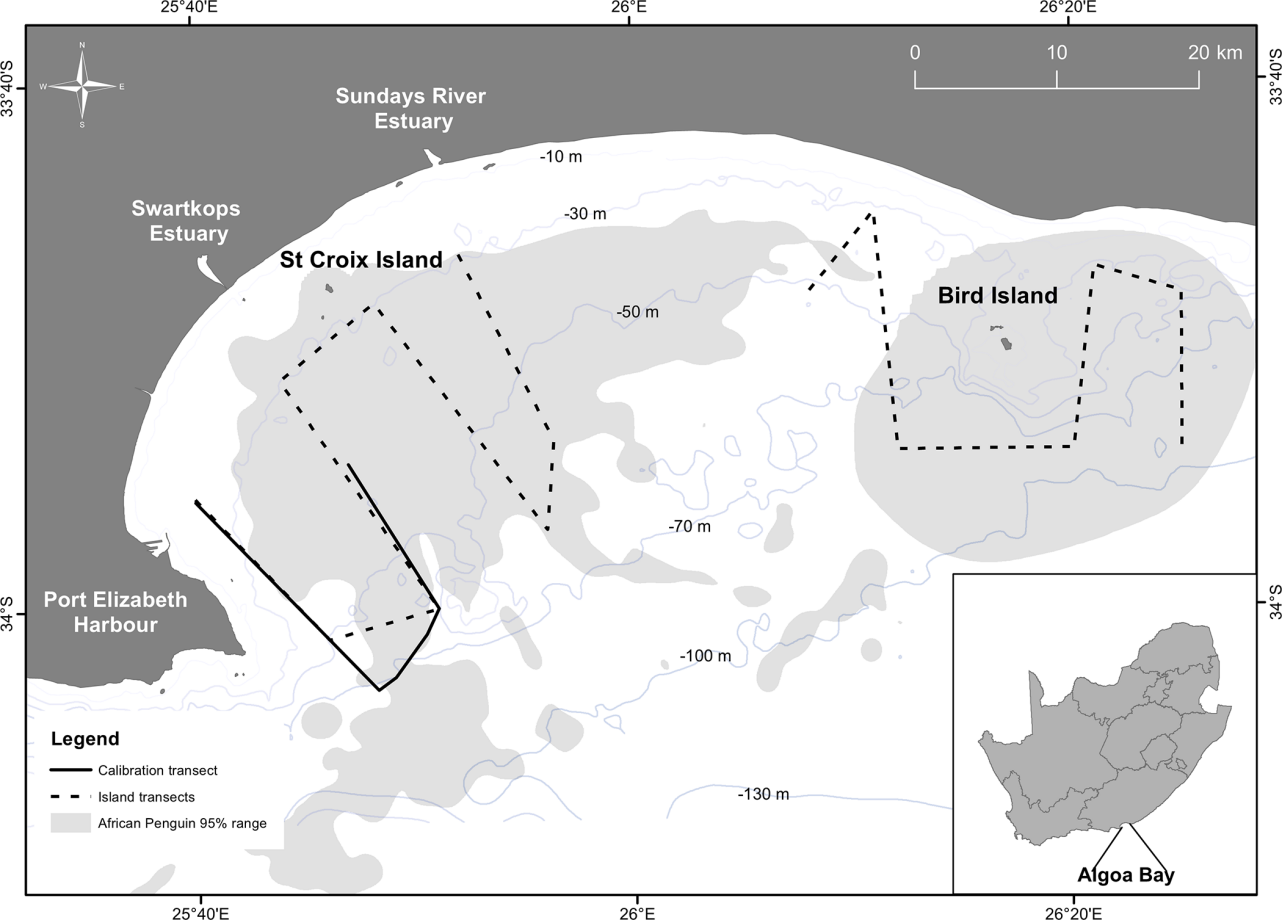
Map of study area showing regular pelagic survey transect routes (dashed lines) around the African Penguin colonies on Bird and St Croix islands and calibration survey route (solid line). Shaded areas denote the 95% kernel density foraging range of African Penguins provisioning small chicks on St Croix and Bird islands between 2008 and 2011 (extracted from [20]).

The inter-calibration was performed along a section of a predetermined survey track (Fig 3) over a distance of 20 nautical miles in water 15–85 m deep. Weather and ocean conditions were calm with <5 knot winds and swell <2 m. The survey was conducted at a speed of 7 knots and was completed in 3 h. Permission for conducting research in Algoa Bay was granted by the South African Department of Environmental Affairs and South African National Parks.

### Data extraction

All echo-returns from both systems were processed through hydoacoustic data processing software, Myriax Echoview 5 for the SES system, and FISH software for the RFF. Once all data were processed they were filtered to minimise the inclusion of backscattering noise and non-fish-school data with the following exclusion rules: all echo-returns <3 m depth (within the acoustic nearfield) and <0.5 m altitude (i.e. bathymetric anomalies); candidates (i.e. contiguous echo returns) of less than 1 x 1 m (L x H) (non-schooling fish); a minimum backscattering strength threshold (S_v_) = –65 dB (following matching between the SES and RFF as explained below). To account for the patchy nature of schools we applied aggregation rules following [26] and [27] to all candidate targets: a minimum linking ellipse of 10 x 2 m (L x H) was chosen as the aggregator and an aggregated school area of 10 x 5 m (L x H) was chosen as the minimum school size.

### Data preparation and statistical approach

#### Comparing FISH and Echoview outputs

Acoustical terminology follows [28]. Due to the non-concordance in overlapping beams of both transducers and the subsequent inability of both systems to completely insonify the same schools, we identified a subset of candidate schools that were most similar in terms of their location and basic morphometric appearance through visual inspection, hereafter termed ‘matched’ schools.

Least squares linear regressions were used where SES outputs were regressed against RFF outputs for the following school parameters from the matched schools: mean school depth (m) and the log transformed school area (2-dimensional cross-section) (m^2^) of the echo-trace.

To allocate energy values to the different pixel colours of the RFF schools we selected three matched schools from the RFF outputs that best represented the full range of pixel variation in our sample by calculating pixel skewness for all matched schools and selecting the three schools with the minimum, closest to zero and maximum skewness values, respectively. For these three scenarios we calculated the mean backscattering strength (S_v_) for all combinations of starting values (i.e. the minimum dB values) between -70 dB and -60 dB (0.5 dB increments) and colour step values of between 0.1 and 2 dB (0.1 dB increments). Sensitivity of these adjustments were assessed against the difference in S_v_ values between the SES outcome for each of these three schools and the corresponding RFF values to isolate combinations in starting values and colour step adjustments with S_v_ differences closest to zero. To correct for saturation in the RFF system (i.e. smaller dynamic range) we applied a correction factor based on the relationship between the mean backscattering coefficients ($\bar{s_{v}}$) of both systems for the three above-mentioned scenarios using the optimal starting and colour step values utilising least squares linear regression techniques. The coefficients of these models were used to predict corrected $\bar{s_{v}}$ estimates for the RFF schools. A log_10_ transformation was applied to the SES $\bar{s_{v}}$ values (i.e. the response variable) due to the exponential relationship between both systems' $\bar{s_{v}}$ values. The corrected $\bar{s_{v}}$ values for the matched RFF schools were then converted into the logarithmic form S_v_ and aggregated into 4 dB bins to compare the frequency of S_v_ values for all three scenarios between both systems before and after application of the correction factor. Wilcoxon signed rank sum tests were applied to the paired S_v_ values of the matched schools between both systems to test if the shape of the distributions of S_v_ values between both systems were significantly different with and without application of the correction factor to the RFF schools.

The potential for time-varied gain (TVG) influences on the attenuation of signal strength with increasing depth in the RFF was checked by regressing $\bar{s_{v}}$ of the matched schools against mean school depth and comparing this relationship with the same schools as derived from the SES. A log_10_ transformation was applied to the $\bar{s_{v}}$ values from both systems' schools to scale the responses to comparative estimates of density.

#### Comparing estimates of relative abundance

To compare estimates of relative abundance from both systems, we calculated the nautical area scattering coefficient (s_A_) (m^2^ nmi^-2^) of all schools aggregated into 500 m Elementary Distance Sampling Units (EDSU), given the formula:

$$s_{A=}4\pi {\left(1852\right)}^{2}s_{a},$$ (1)

where 4π(1852)^2^ is the nautical mile derived scaling factor and s_a_ is the integral of $\bar{s_{v}}$ over a range interval, following [28]. In the context of this study, the range interval is the height of all schools weighted by the length of all schools for schooling fish targets only.

A logistic regression model was used to determine the relationship between school encounters (fish present = 1, fish absent = 0) in the 500 m EDSUs by both systems. To determine the scale at which s_A_ estimates were most concordant between both systems we generated rolling sums of s_A_ values between scales of 0.5 km and 18 km using the R package ‘zoo’ [29]. This was achieved by summing all s_A_ values over the rolling window and dividing this value by the scale length. Least squares linear regression models were used to compare the coefficients of determination (R^2^) of the relationships between s_A_ values of both systems at 18 different scales within this range. We applied logtransformations to both variables after adding 0.1 to account for EDSUs with no schools recorded. After transformations both variables were approximately bell-shaped.

All statistical analyses as well as graph outputs were completed in the statistical package R [30].

## Results

### Comparing FISH and Echoview outputs

**Fig 4:**
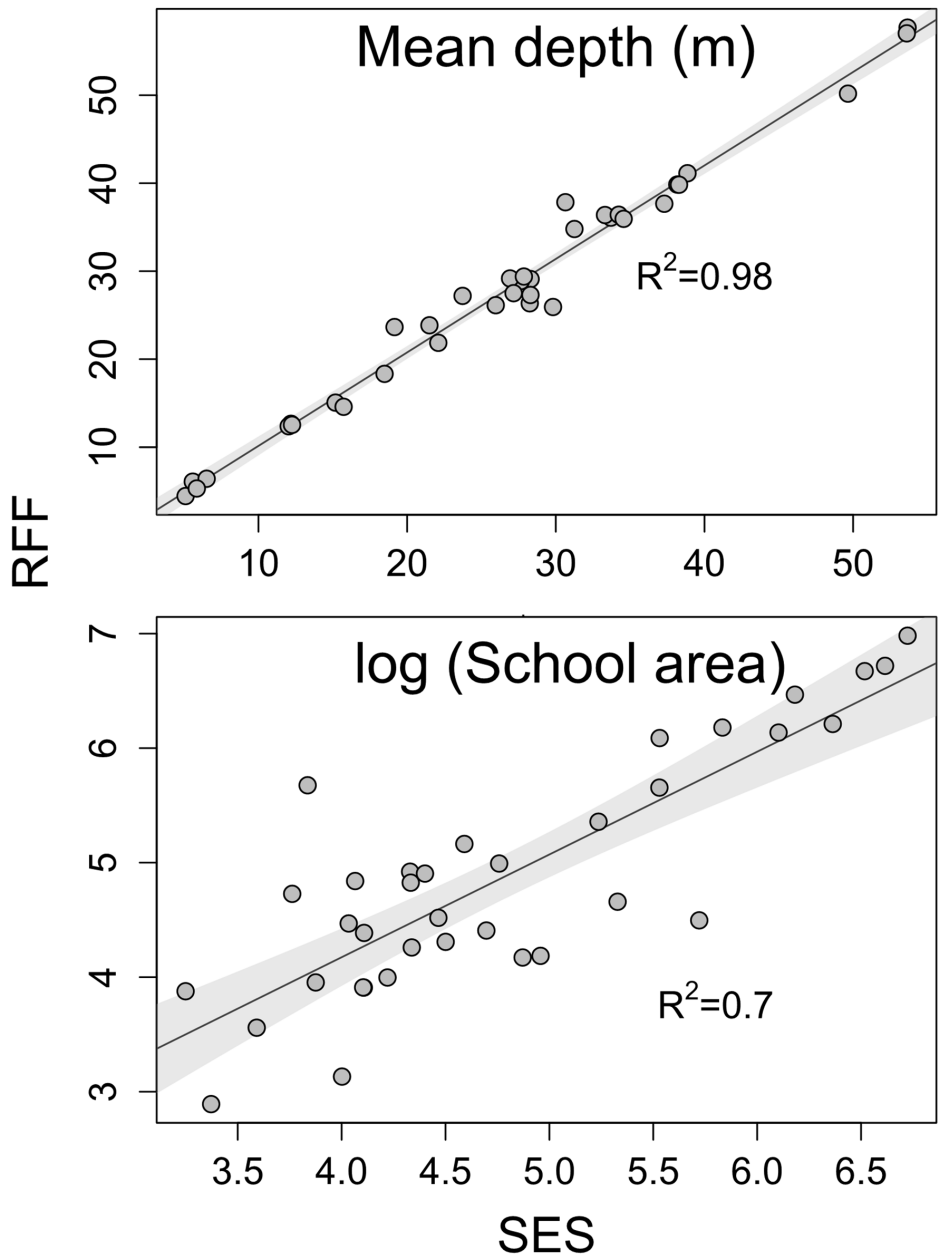
Linear regressions showing the relationships between school desciptors, mean depth (top graph) and log transformed school area (bottom graph), for 36 matched schools from the recreational fish-finder (RFF) and the scientifc echo-sounder (SES). Shaded areas denote 95% confidence intervals.

After applying the filtering procedures, 93 schools were insonified by both the RFF and the SES, of which 36 schools (38%) were classified as matched and were used for comparing the two systems. Estimates of mean school depth between matched schools from both systems were highly significant (R^2^ = 0.98, Fig 4), as were estimates of school area (R^2^ = 0.70, Fig 4). Estimates of mean school depth ranged from 5.1 to 53.7 m (mean±SD = 26.5 ± 12.6 m) and 4.5 to 57.7 m (27.6 ± 13.6 m) for the SES and RFF, respectively. Estimates of school area ranged from 25.8 to 831.9 m^2^ (195.7 ± 217.9 m^2^) and 18 to 1076 m^2^ (225.4 ± 260.3 m^2^) for the SES and RFF, respectively.

### Density estimates and applying the correction transformation

**Fig 5:**
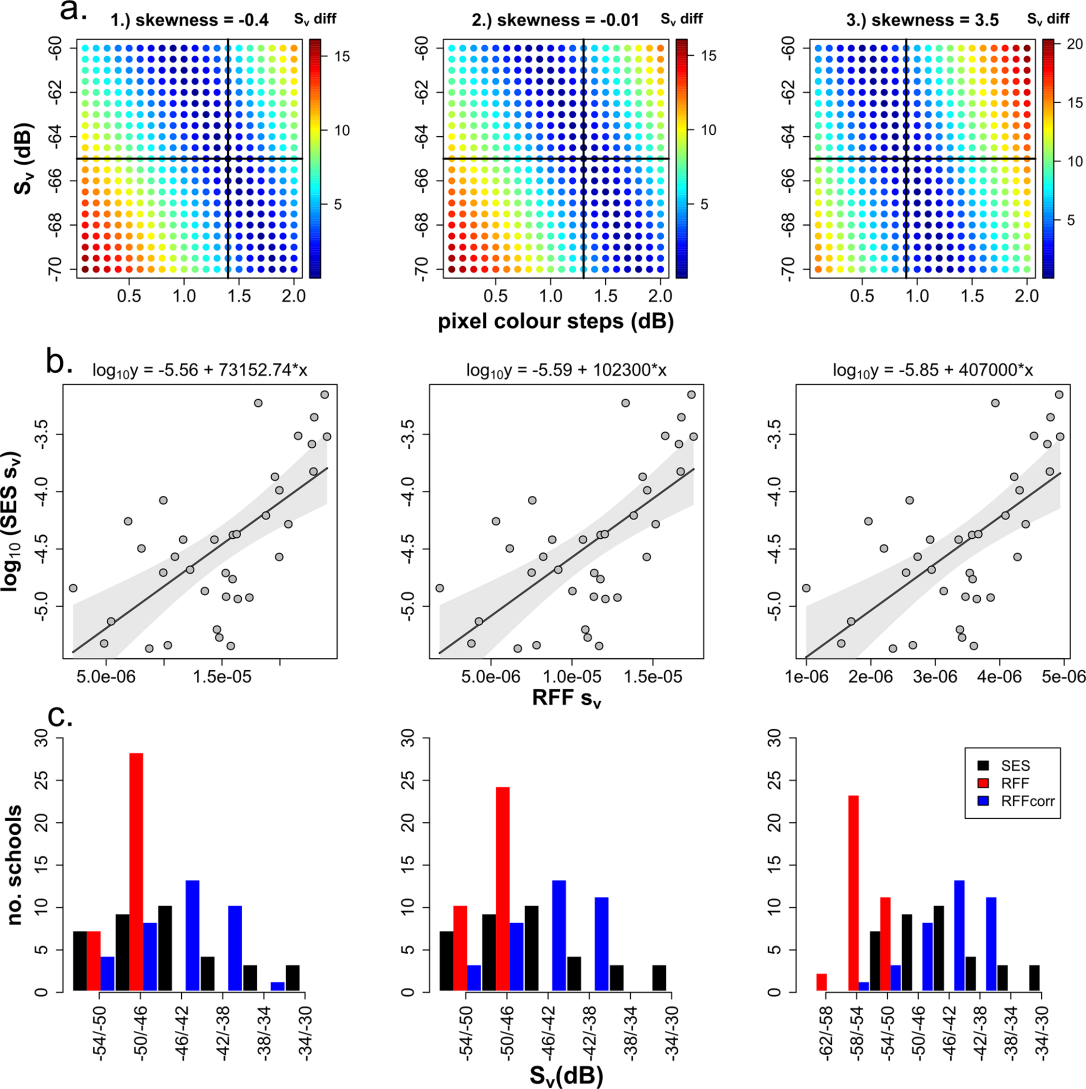
Plots for three scenarios, 1–3, representing three schools selected from the recreational fish-finder (RFF) outputs with different pixel skewness values: a) 2D scatter plots showing the influence of different combinations of mean volume backscattering strength (Sv) starting values and colour step values on the difference in Sv values (Sv diff) between the RFF and the scientific echosounder (SES) outputs (colour scale bar—low values indicate optimal estimates), cross-hatch denotes optimal colour step values at starting values of -65 dB; b) least-squares regressions between volume backscattering coefficients ($\bar{s_{v}}$) of the SES (transformed) and RFF outputs of the 36 matched schools using starting and colour step values for the RFF outputs as determined by the 2D scatter plot analyses, coefficients are given at the top of each plot and shaded areas denote the 95% confidence intervals; c) histograms showing the frequency of schools in 4 dB S_v_ bins for the SES-derived schools, the RFF-derived schools before application of a correction factor as predicted by the linear regression models (b), and the RFF-derived schools with this correction factor applied (RFF corr).

The three matched schools selected to determine optimal starting and colour step dB increments for the RFF pixel values had skewness values of -0.4, -0.001 and 3.5 representing the matched schools with the lowest pixel colour values (i.e. left skewed), the most moderately skewed pixel values (i.e. closest to 0), and the most saturated pixel values (i.e. right skewed), respectively. The differences in S_v_ values between SES and RFF for all starting and colour step combinations for these three scenarios are illustrated in Fig 5A. For all three scenarios there was a negative linear relationship between Sv differences with starting values and optimal colour step values (i.e. as shown by the dark blue points in Fig 5A) with the centrally located starting values showing a good spread in the distribution of optimal starting values. Based on these results a S_v_ of -65 dB was selected as the baseline starting value to compare different corresponding optimal colour step values in the three different scenarios; this was deemed appropriate to facilitate comparisons with the SES starting values, i.e. also -65 dB. The optimal colour step values at this baseline starting point were 1.4, 1.3 and 0.9 dB for the three school scenarios 1–3, respectively, and these values were mapped to the 36 matched schools in each case.

Results of the least squares linear regressions between the $\bar{s_{v}}$ values of both systems with the new mapped pixel values for the RFF schools are shown in Fig 5B. All three scenarios have almost identical positive linear trends with very similar intercepts but varying slope coefficients. Applying these model predictions as a correction to the RFF S_v_ values and comparing binned S_v_ values between systems before and after correction showed improvements in dynamic range for all three cases, especially scenarios 1 and 2 (Fig 5C). This was evident in the medians and variances in the corrected school S_v_ values as well as the improvements in Wilxon signed ranked sum test probabilities (Table 3). Despite this improvement, the concordance in dynamic range after correction was still limited in the RFF system outputs especially for schools with higher densities (Fig 5C, Table 3).

**Table 3:** Summary statistics of mean volume backscattering strength (S_v_) estimates for 36 matched schools as insonified by the scientific echosounder (SES) and the recreational fish-finder (RFF) using outputs for three scenarios representing different pixel derived outputs for the RFF system: IQR—interquartile range, p—Wilcoxon signed rank statistic probability estimates between the pairs of schools derived from different system outputs, i.e. between all RFF scenarios and the SES outputs.

	Scenario	dBi	min	max	median	IQR	p
SES Sv		1	-53.7	-31.5	-45.3	8.6	
RFF Sv	1	1.4	-56.7	-46.2	-48.1	2.6	0.0002
RFF corrected Sv	1	1.4	-53.9	-37.9	-44.2	6.5	0.94
RFF Sv	2	1.3	-57.4	-47.6	-49.4	2.5	2.05E-006
RFF corrected Sv	2	1.3	-54.1	-38	-44.2	6.5	0.94
RFF Sv	3	0.9	-60	-53.1	-54.5	2	2.91E-011
RFF corrected Sv	3	0.9	-54.4	-38.4	-44	6.2	0.94

**Fig 6:**
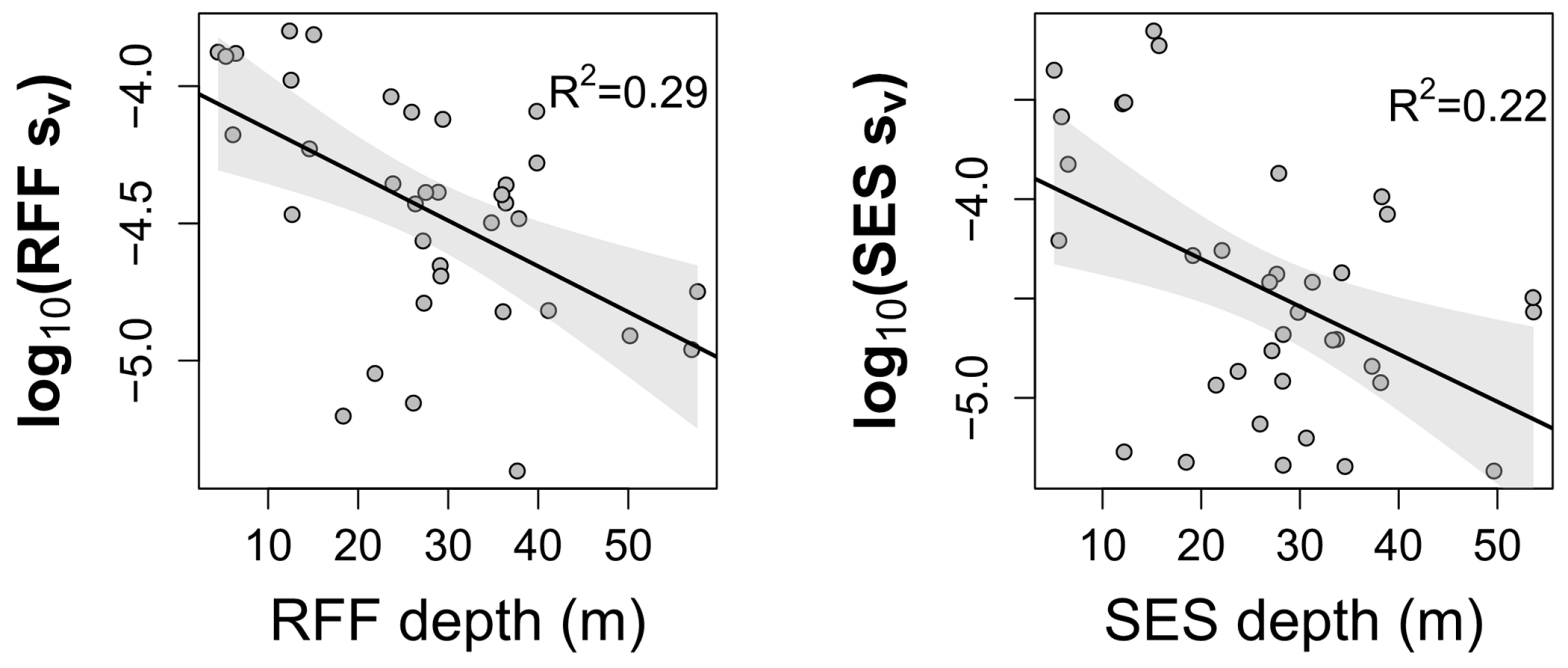
Least squares linear regressions showing the relationship between volume backscattering coefficients ($\bar{s_{v}}$) and mean school depth for the 36 matched schools insonified by the recreational fish-finder (RFF) and the scientific echosounder (SES). Shaded areas denote 95% confidence intervals.

Comparisons of the influence of depth on $\bar{s_{v}}$ estimates, i.e. the potential influence of TVG, for the RFF and SES systems are shown in Fig 6. Both systems showed similar negative trends with $\bar{s_{v}}$ values decreasing with increased depth but these relationships were weak explaining 29% and 22% of the variation in the RFF and SES comparisons, respectively.

### Comparing estimates of relative abundance

**Fig 7:**
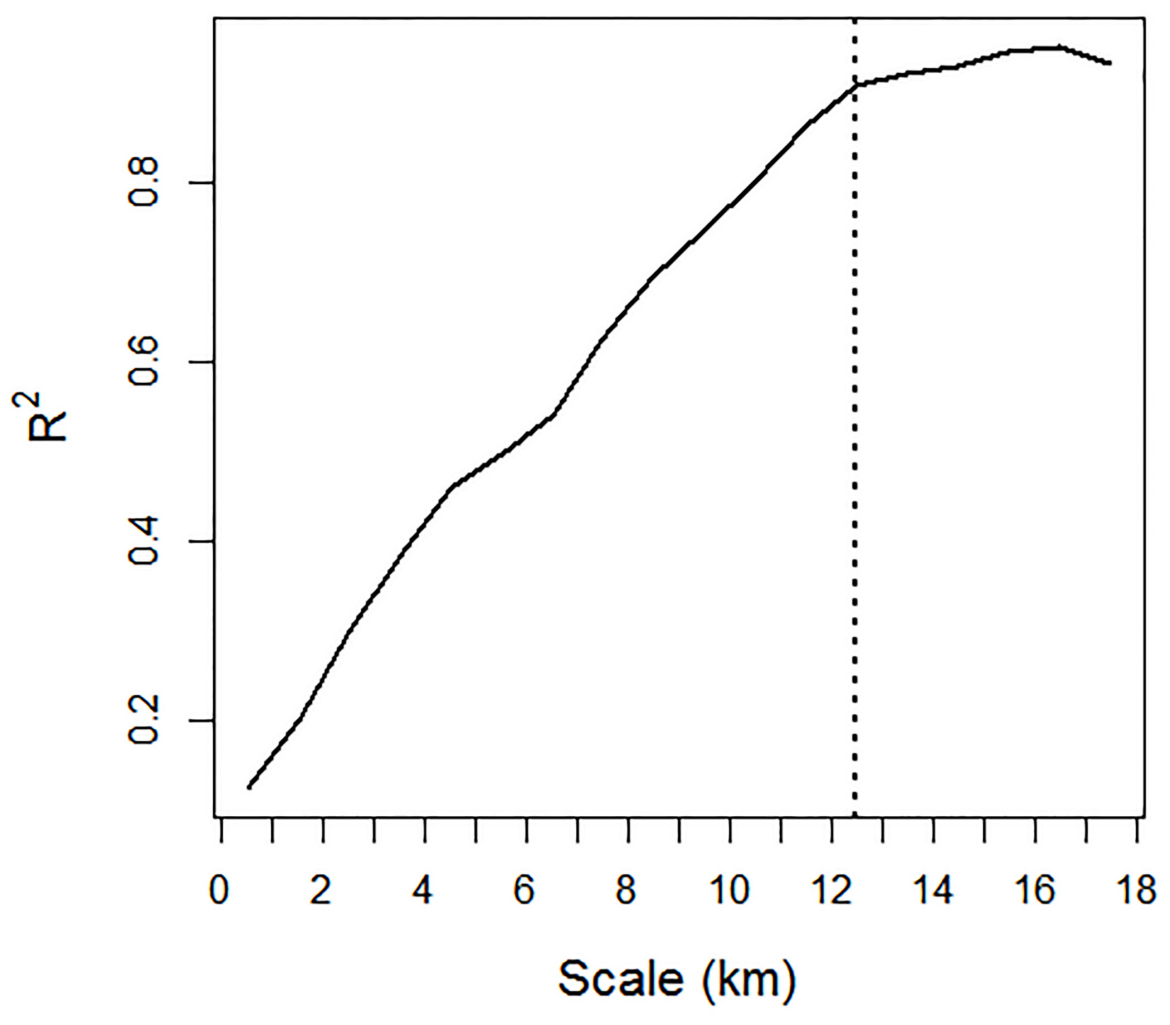
Coefficient of determination (R^2^) values of least squares regressions for 18 models comparing relative abundance estimates using the nautical area scattering coefficients (s_A_) (m^2^ nmi^-2^) between schools from the scientific echo-sounder (SES) and the recreational fish-finder (RFF) at different scales: 0.5 km–18 km, hatched vertical line denotes asymptote (12.5 km).

Table 4 shows the detection frequency of insonified schools by both systems within 500 m EDSUs. In the majority (67%) of the 76 EDSUs, both systems recorded either the presence (37%) or absence (30%) of schools, concurrently. The RFF detected schools in ten of the EDSUs where the SES failed to detect any schools and the SES detected fish schools in 15 instances where the RFF failed to detect any schools. Results of the logistic regression showed a significant relationship between the concurrent detectability of both systems (P < 0.01). We used s_A_ as a measure of relative abundance using the corrected $\bar{s_{v}}$ values as determined from scenario 2 (Fig 5), i.e. S_v_ starting values of -65 dB and colour steps of 1.3 dB. Comparisons of these values aggregated for each 500 m EDSU showed no significant differences in these estimates between systems: RFF (median, interquartile range = 17.3, 532.9 m^2^ nmi^-2^); SES (16.1, 279.3 m^2^ nmi^-2^), Wilcoxon rank sum test (P = 0.9). Comparisons of relative abundance estimates at different scales are illustrated in Fig 7. There is a clear positive linear trend in the relationship between model fit estimates (R^2^) and scale reaching an asymptote at approximately 12.5 km at which point this particular scale explains 91% of the variation between systems.

**Table 4:** Frequency of fish school encounter scenarios of the recreational fish-finder (RFF) and the scientific echo-sounder (SES) quantified by 500 m Elementary Distance Sampling Units (EDSU).

RFF	SES	No. EDSUs
fish	fish	28
fish	no fish	10
no fish	fish	15
no fish	no fish	23

## Discussion

The small proportion (39%) of matched schools compared to the total number of schools insonified by both systems during the calibration survey can be attributed to the athwartship displacement of the transducers (2 m) (Fig 2) and the inability to synchronise the pings between both transducers. These differences are known to influence the comparability of school parameters insonified by different echo-sounders used on the same vessel, although it is often impractical to avoid these sources of error [31]. A combination of these factors is likely to have some bearing on the unexplained variation in the associations between $\bar{s_{v}}$ values between the matched schools (Fig 5B). However, the limited dynamic range of RFF systems is likely to have had a larger influence on this variation, especially for higher density schools. The correction factors applied to the RFF schools improved the dynamic range for all three pixel substitute scenarios although these were still limited for schools with higher densities and we caution against using these devices for accurate measures of abundance such as is possible with more sophisticated SES technology. Notwithstanding these limitations, relative abundance estimates using all schools from both systems showed strong congruence especially at scales > 10 km and the RFF matched the SES system in terms of school detectability, a potential proxy for relative abundance estimates [32]. Comparisons of potential influences of TVG (i.e. depth dependencies on energy values) showed little evidence for this effect in the RFF outputs. This is because both systems showed similar weak, negative associations between depth and $\bar{s_{v}}$ estimates despite the SES system having been calibrated to ameliorate this source of error. These trends are more likely to have been influenced by factors other than TVG, such as sound attenuation that is known to occur in high density schools [33] and the diverging depth dependencies of different fish species, notably round herring (*Etrumeus whiteheadi*) which occupy deeper depths than other forage fish species during the day in this region [27,34].

Despite large differences in the fabrication of the two echo-sounder systems, their use, specifications and costs, results of the inter-calibration survey, as quantified by the FISH programme, demonstrate the ability of a Furuno RFF to produce comparable outputs to the SES used in this assessment. This is strongly encouraging for marine ecology studies that require estimates of prey distribution and relative abundance but lack the budget or expertise to use SES systems.

Our study has demonstrated the ability of the FISH programme to extract accurate estimates of school depth and size (i.e. school area). These parameters can provide valuable inputs into marine ecology studies. For instance, the vertical location of prey is significant in terms of its accessibility in relation to a predators maximum and optimal dive depths [35,36] and school depth relative to the seabed (i.e. school altitude) is likely to affect predators that pursue their prey from below, e.g. baleen whales [37], seals [38] and penguins [39,40]. School depth and altitude data have proven to be effective acoustic determinants of pelagic fish species identification in South Africa, especially when coupled with ancillary data (i.e. location, sea surface temperature and time of day) although the use of this information needs to be calibrated for the region of interest and the period during which the surveys take place [27]. The frequency and distribution of schools of different sizes can be used to test hypothesis related to school encounter and detectability rates and hunting success. For example, [41] inferred the tendency of African Penguins to target small schools of anchovy and postulated the benefits in terms of increased encounter rates when compared to larger more patchy schools. We have conducted simultaneous bio-logger deployments of African Penguins and pelagic fish surveys to further explore and test some of the above-mentioned hypotheses using school descriptors derived from our RFF.

The efficacy of using seabirds as indicators of ecosystem function and in informing marine conservation management depends on the predictive power of the various ecological parameters that can be harnessed from these species [11]. Activity budgets and breeding parameters (e.g. colony attendance and chick growth rates) provide a convenient yardstick to infer variation in the marine prey base. However, informative thresholds of prey yield are confounded by these species’ ability to adapt to variability in food supply (e.g. [42,43]). Functional relationships between seabird behaviour parameters and prey quantity, as originally hypothesised by [7], are typically curvilinear, the position of the informative ‘tipping points’ being dependent on the influence of the inherent behavioural plasticity on the parameter used (e.g. [44]). Simultaneous data on prey availability is essential if these thresholds are to be determined and it is only recently that empirical studies of this nature have been conducted. [45] determined these relationships by simultaneously measuring prey abundance while recording breeding parameters of Common Murres (*Uria aalge*). Their findings reflected [7] non-linear response predictions providing quantifiably more meaningful determinants of ecosystem change. These relationships need to be determined for each potential indicator species and the missing element in realising these is often concurrent prey data.

An important focus of our research into the impacts of purse-seine fishing on African Penguins is teasing out the natural fluctuation in prey abundance from the effects of fishing. Prior to regularly surveying fish distribution and abundance in the foraging area of breeding penguins in 2012 (Fig 3) using the techniques described in this paper, most data on pelagic fish abundance was collected from annual stock assessment surveys conducted over large spatial scales by DAFF. These were typically undertaken in November of each year, after African Penguins had ceased breeding. In 2009, the first of the six-year island closure experiments was implemented around St Croix Island, the world’s largest African Penguin breeding colony [21]. Comparative results of penguin foraging effort parameters, using bio-logger technology, before and after the closures, showed significant differences in the amount of effort these birds put into their at-sea activities [20]. As alluded to previously, the efficacy of these results were undermined by a lack of data on the natural variability in the prey base. Information gathered from RFFs can be used to offset this shortfall.

## Conclusions

The results of this study apply specifically to the Furuno RFF system used and the context within which this system was operated, i.e. in-shore forage fish species in the Benguela Upwelling Region. Prospective users of these systems need to weigh the merits of adopting such an approach against the circumstances of their particular study. Some key considerations include: the programming capacity to modify the FISH software to different RFF models; the species targeted for and the ability of the RFF to quantify meaningful parameters of these targets; the depth range of the RFF, and; access to a SES and technical expertise for calibration purposes. We have attempted here to give context to the colour scale display of the RFF and the outputs derived are broadly comparable with those obtained from the SES at scales relevant for marine predator-prey interaction studies. This should allow for meaningful comparisons of relative fish density and biomass and school descriptors within surveys conducted using RFFs. However, estimates of fish abundance derived from such systems should be used with caution given the inability to calibrate RFFs and monitor their performance over time. Provided there is no large drift in the performance of the RFF over time, comparisons of fish school parameters between surveys should be possible. The development of hydroacoustic data processing software for RFF echo returns (FISH) greatly facilitates data capture, and can be modified for use with echo returns from other RFF models. The programme and its source code are available from http://www.cbio.uct.ac.za/~arjun/. The methods outlined in this study can be adapted to a broad range of marine top predator studies that utilise boat-based survey or observation techniques.

To facilitate the adoption of this method by other users we have provided supplementary material including a summarised guide (S1 File) outlining the steps taken to perform these types of calibration surveys. The R code to perform the pixel calibration and subsequent analyses as shown in Fig 5 is provided (S2 File) as well as the data used in all analyses (S3–S6 Files).

## Supporting Information

S1 File Summarised guide to recreational fish-finder (RFF) calibration procedures, mapping of energy values to pixel colours and performing the energy correction transformation. (DOCX)

S2 File R code for mapping pixel values and performing the correction factor. (TXT)

S3 File csv data file—All school outputs for the SES system. (CSV)

S4 File csv data file—All school outputs for the RFF system. (CSV)

S5 File csv data file—Matched dB values file for use with S2 R code. (CSV)

S6 File csv data file—Matched school parameters for both systems, 36 schools. (CSV)
