# Supplementary material for: Recreational Fish-Finders—An Inexpensive Alternative to Scientific Echo-Sounders for Unravelling the Links between Marine Top Predators and Their Prey
Source: PLoS One. 2015 Nov 23;10(11):e0140936. doi: 10.1371/journal.pone.0140936 (PMC4658084; doi:10.1371/journal.pone.0140936)
Supplement: S1 File — (DOCX) [file pone.0140936.s001.docx]

S1. Summarised guide to recreational fish-finder (RFF) calibration procedures, mapping of energy values to pixel colours and performing the energy correction transformation.

1. Perform a calibration survey with the scientific echosounder (SES) making sure that the following recommendations by Korneliussen et al. (2008) are adhered to as close as is possible. Key considerations:
   1. match beam widths;
   2. mount transducers as close as is possible;
   3. if possible, synchronise pulse duration;
   4. synchronise time stamps.
2. Ensure that a sufficient sample of schools is insonified for the purposes of identifying an adequate sample of matched schools and estimating relative abundance at different spatial scales.
3. Identify the most complete segments of schools insonified by both transducers, i.e. matched schools through visual inspection of both outputs.
4. Calculate $\bar{s_{v}}$ values for matched schools of the SES (using e.g. Echoview software).
5. Identify candidate matched schools of the RFF system that best represent a good diversity in pixel spread - skewness values can be used to select these objectively.
6. Using the R script provided in S2, generate a matrix comparison (Figure 5a) for the different pixel spread scenarios.
7. Identify optimum starting and increment dB values from the graph outputs looking at values that correspond to a S_v_ difference closest to 0 ( as with Figure 5a shown in dark blue).
8. Map these values to the matched RFF schools and calculate $\bar{s_{v}}$ . These are the uncorrected estimates and the code to map these values and calculate $\bar{s_{v}}$ are provided in S2.
9. Model the relationship between $\bar{s_{v}}$ values of the SES (response) and the RFF for all matched school outputs. The modelling approach will depend on the nature of the data and will therefore be user specific.
10. Use this model to predict corrected $\bar{s_{v}}$ values for the RFF schools and explore the efficacy of this correction by plotting the dynamic range, e.g by using S_v_ binned values (Figure 5c) before and after application of the correction factor. The R code for our example is provided in S2.
